# Towards the Design of a Patient-Specific Virtual Tumour

**DOI:** 10.1155/2016/7851789

**Published:** 2016-12-19

**Authors:** Flavien Caraguel, Anne-Cécile Lesart, François Estève, Boudewijn van der Sanden, Angélique Stéphanou

**Affiliations:** ^1^Clinatec, INSERM UA01, 38054 Grenoble, France; ^2^Université Grenoble Alpes, CNRS, TIMC-IMAG/DyCTIM2, 38041 Grenoble, France; ^3^Université Grenoble Alpes, EA 7442 RSRM, ID17-ESRF, 38000 Grenoble, France

## Abstract

The design of a patient-specific virtual tumour is an important step towards Personalized Medicine. However this requires to capture the description of many key events of tumour development, including angiogenesis, matrix remodelling, hypoxia, and cell state heterogeneity that will all influence the tumour growth kinetics and degree of tumour invasiveness. To that end, an integrated hybrid and multiscale approach has been developed based on data acquired on a preclinical mouse model as a proof of concept. Fluorescence imaging is exploited to build case-specific virtual tumours. Numerical simulations show that the virtual tumour matches the characteristics and spatiotemporal evolution of its real counterpart. We achieved this by combining image analysis and physiological modelling to accurately described the evolution of different tumour cases over a month. The development of such models is essential since a dedicated virtual tumour would be the perfect tool to identify the optimum therapeutic strategies that would make Personalized Medicine truly reachable and achievable.

## 1. Introduction

At the State of the Union address 2015, President Obama launched the* Precision Medicine Initiative* (https://www.whitehouse.gov/precision-medicine) with cancer and diabetes as the main targets [1]. Precision medicine is an innovative approach that takes into account patient variability so that treatments are tailored to patient-specific characteristics, mainly the genetic profile. This aims to significantly improve the treatment efficacy and chances of survival. Precision medicine, not to be confused with* Personalized Medicine*, is thus based on the identification of common characteristics in patient subpopulations. That means that treatment is adapted to specific subclasses but cannot be, per se, individualized as Personalized Medicine aims it to be [2]. Classification of subgroups of patients relies on processing a large amount of data, big enough to be reliable and to help in confident decision making. The rise of big data over the recent years has paved the path for this type of approach and its application to medicine [3–6]. Although we can expect significant progress from it, it still has some severe drawbacks already pointed out by Mi et al., (2010) [7]. First, precision medicine, in the current stage of research, mostly relies on genetic profiling. However, it is now clear that genetic knowledge alone is not sufficient to predict the evolution of a disease such as cancer for which environmental conditions can affect genetics indirectly by modifying epigenetic factors from the cell to the tissue scale [8, 9]. Second, data analysis performed for genetic profiling is essentially a correlative process that brings very little insights for the reasons why treatment is (or would be) efficient or not. Finally, cancer is an evolutive and very heterogeneous disease with many different stages involving temporal variability on the tumour dynamic and on the patient state that cannot be easily predicted. This requires the development of pathophysiological models that integrate the underlying key mechanisms precisely describing the evolution of the disease for predicting and understanding its behaviour and its response to treatment [10–14].

To that end, we developed a computational model that integrates key pathophysiological mechanisms to describe the growth of real tumours observed in a mouse pinna, so as to build an avatar or virtual tumour for each observed case. In order to build an accurate virtual clone, the experimental mouse model was chosen in a way to provide sufficiently detailed microscopic information on the tumour evolution and on its vascular environment. We chose to use immunodeficient nude mice to ensure the growth of the tumour and also in order to neglect the interplay with the immune system. This allows us to simplify the elements to integrate in the computational model in this first stage towards the development of a mouse-specific virtual tumour. As proof of the concept, the virtual tumour evolution should mimic its real life evolution. The growth of seven different tumour cases were virtually described with a good accuracy over a month. Moreover the model was able to capture a characteristic event in the experimental growth process corresponding to the well-known angiogenic switch [15] and that we described as an angiogenic bottleneck. This marks the progressive transition between avascular and vascular tumour growth at a specific time. This study shows that the virtualization of a patient's tumour is achievable using medical imaging techniques for the measurements of pathophysiological parameters (*i.e.*, tumour size, shape, density, and vascular configuration) to be able to define, in a near future, truly personalized and optimized treatments.

## 2. Materials and Methods

### 2.1. The Experimental Model

#### 2.1.1. Animal Model

We used a mouse ear tumour model which consists in the injection of tumour cells in the dermis of mice pinna [16]. This minimally invasive model allows following the development of the tumour and of its vasculature over a long time period. In accordance with the policy of Clinatec and the French legislation, experiments were done in compliance with the European Parliament and the Council of the European Union Directive of September 22, 2010 (2010/63/EU). The research involving animals was authorized by the Ministère de l'Enseignement Supérieur et de la Recherche. For the experiments, female athymic Nude-  Foxn1^nu^ mice were used. Mice were housed in ventilated cages with food and water ad libitum in a 12 h light/dark cycle at 26 ± 1°C. For in vivo imaging or injections, mice were anaesthetized using isoflurane (5% for induction and 2% during the experiment) in a 80% air and 20% O_2_ gas mixture.

#### 2.1.2. Cell Culture

The U87-MG cell line (primary human glioma) was obtained from the American Type Culture Collection (ATCC HTB-14) where cell line authentication and species identification was conducted. The cells were transfected with the Green Fluorescence Protein (GFP). The cells were grown in Dulbecco's modified Eagle's medium (DMEM) containing 10% heat-inactivated foetal bovine serum, 2% L-glutamine, penicillin (100 IU/mL), and streptomycin (100 *μ*g/mL). The cells were kept in standard culture conditions (100% relative humidity, 95% air, and 5% CO_2_). The culture medium was changed twice weekly.

#### 2.1.3. Tumour Generation

Tumour was generated by injecting a 2 *μ*L solution of 2 · 10^5^ U87-GFP MG cells in HC Matrigel (Corning, New York, United States) in mouse ear dermis. During the whole injection procedure, cells and cells/matrigel solution were kept on ice. Prior to injection, anaesthesia was performed and ears were taped to a conical tube for easy injection of the tumour cells using a 26-gauge custom needle mounted on a RN-701 Hamilton syringe. Immediately after injection the presence of tumour cells was controlled by fluorescence microscopy.

#### 2.1.4. Experimental Setup

The ear was gently placed in a custom-built ear holder and immobilized under a coverslip with ultrasonic gel in between the ear and the coverslip. Body temperature was monitored with a rectal probe and maintained at 36°C during the whole imaging session using a heating pad with feedback. Acquisitions were performed only when body temperature reached 36°C in order to avoid hypothermia and vascular constriction during anaesthesia.

#### 2.1.5. Microscopy

**Figure 1:**
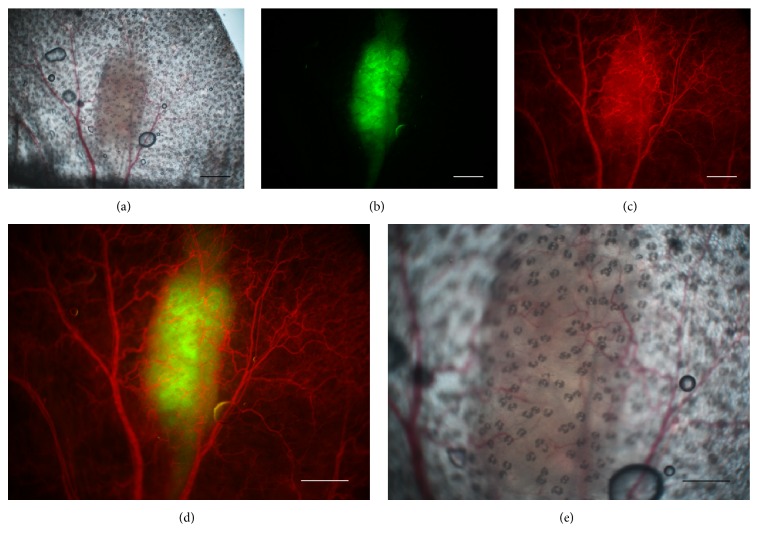
Imaging modes. Bright field and fluorescence imaging of mouse pinna bearing a glioblastoma tumour are used to follow the tumour and vascular evolutions simultaneously. Images were here acquired 14 days after cells injection (case M1-R). (a) Bright field image of the tumour and vasculature, (b) fluorescence image of U87-GFP tumour cells, (c) blood vasculature highlighted by dextran-rhodamine, (d) the two fluorescent channels which are merged, and (e) bright field image zoom on the tumour and microvasculature with objective 4x. All other images have been acquired with objective 2x. Scale bars: (a), (b), (c), and (d) 1 mm; (e) 500 *μ*m.

Tumour imaging was performed twice a week during one month using a Nikon AZ100 multizoom microscope (Nikon France, Champigny-sur-Marne, France), equipped with 1x (0.1 NA), 2x (0.2 NA), and 4x (0.4 NA) objectives. Fluorescence and bright field imaging was performed for each tumour (Figure 1) with NIS-Element software package. The vascular network was highlighted in red fluorescence by injecting 100 *μ*L of a 20 mg/mL solution of Rhodamine B isothiocyanate-dextran (Sigma Aldrich) in the vein of the mouse tail on days 7 and 14.

#### 2.1.6. Image Analysis

Image analysis was performed using ImageJ (version 1.47). To monitor apparent tumour growth, a grey-level threshold was applied on the GFP-images. Yen's thresholding method was used [23] and manually adjusted to correct some artefacts. The more restrictive Default ImageJ filter was also used as a comparison. The area of the tumour was then measured from the filtered image.

#### 2.1.7. Tumour Identification

The results presented correspond to experiments made on 4 mice bearing two tumours, one on each ear. Each mouse (M) is assigned a number (*X*) and the tumour is identified as left (L) or right (R). The different tumours are thus coded M*X*-L/R (with *X* from 1 to 4).

### 2.2. The Computational Model

#### 2.2.1. Cell States

A cellular automaton, under the form of a square grid, is used to describe tumour growth and the evolution of the tumour cells state. Full details are available in [17, 24]. Transitions between four possible states are considered: proliferative, quiescent, apoptotic, and necrotic, denoted as *P*, *Q*, *A* and *N*, respectively. The default state for a normal (physiological) level of oxygen is the proliferative state. If the level of oxygen decreases below a threshold and the cell is in an oxygen-sensitive phase, which is assumed to be restricted to the G1-phase of its cycle, then the cell becomes quiescent [25]. It can reverse to the proliferative state if the oxygen level comes back to normal or above. If the level of oxygen becomes too low then the cell dies through necrosis [26].

#### 2.2.2. Cell Cycle and Cell Division

The duration of the cycle of a cell after division (daughter cell) is assumed to be slightly different from this of the dividing cell (mother cell). It is determined using a truncated Gaussian distribution centred on the duration (*μ*) of the cycle of the mother cell with standard deviation *σ* = 0.2 hours. Only the duration of the G1-phase is assumed to be altered since this phase is known to vary the most between cells coming from a same clone [27]. In the automaton, the cell can only divide if there is some available space, that is, (i) if there is a free element among the 8 neighbour elements of the square grid or (ii) if the dividing cell can push a neighbour cell in a free element beyond the first row of occupied elements; if not the cell enters apoptosis [28].

#### 2.2.3. The Vascular Network

Based on the results from a previous study [17], the capillary, microvascular, and angiogenic networks are differentiated and modelled differently. First the capillary network is represented under the form of an implicit submicrovascular field with *E*_*i*,*j*_ = *E*_0_ at each point of the simulation grid (*i*, *j*) where *E*_0_ corresponds to the normal density of capillaries which ensures a physiological ground level of oxygen. In the model, it can be locally degraded (*i.e., E* = 0) by proteases produced by proliferating tumour cells [29, 30]. Shortage of oxygen can thus occur and influence the tumour evolution in many ways that we limit here to cell cycle arrest. Second, the microvascular network consists of vessels with diameters ranging from 30 to a few hundreds micrometers (capillaries and neovessels are thus excluded). The vessels are mapped from the experimental images and introduced into the automaton. Third, the angiogenic network corresponds to the newly formed vessels sprouting from the microvascular network. The microvascular vessels and neovessels can occupy the edges and diagonals of the grid elements where the length of a vessel is *L*_*b*_ = Δ*x*, if the vessel is on the edge (border) of the element and $L_{d}=\Delta x\sqrt{2}$, if it lies on one of the diagonals. For each grid element (*i*, *j*) the* vessels weight W*_*i*,*j*_ can be calculated to evaluate the contribution of the neovessels *v* in providing oxygen or in consuming growth factors:

(1) $$\begin{array}{c} W_{i,j}=\underset{v\in \left(i,j\right)}{\sum }\left(\;\frac{L_{b}}{2}+L_{d}\;\right)+E_{i,j}. \end{array}$$

We note that the length of the vessels at the border of the element is divided by 2, since the vessels contribute to 2 elements (left/right or up/down). Angiogenesis, that is, the formation of the new vessels, is described as in [17, 18, 24, 31].

#### 2.2.4. The Extracellular Matrix

It evolves when the tumour develops and can play an important role to stabilize the tumour or at the opposite to favour tumour invasion [32]. Here we neglect the matrix fibre production and the role it plays on the tumour. However the model takes into account matrix degradation occurring during angiogenesis* via* the proteases produced by the migrating and proliferating endothelial cells [33].

#### 2.2.5. Diffusive Molecules

Such growth factors and oxygen influence the relationship between tumour growth and vascular growth in a reciprocal way. Growth factors (*V*) mainly produced by hypoxic tumour cells trigger vascular growth through angiogenesis and reciprocally the new vessels provide oxygen (*O*) that fuels the growth of the tumour through cell proliferation. The other diffusive molecules involved in the model are the proteases produced by the proliferating tumour cells (*p*) and by the migrating endothelial cells (*m*) that degrade the capillary network and extracellular matrix fibres, respectively. The equations that rule the spatiotemporal dynamics of the concentrations for all diffusive species {*V*, *O*, *p*, *m*} are given by

(2) ∂V∂t=DV∇2V+αVQi,j−νVV−λVWi,jmin⁡V,Vmax,∂O∂t=DO∇2O+γvWi,jOv−O−ki,jO,∂p∂t=Dp∇2p+αpPi,j−νpp,∂m∂t=Dm∇2m+αmni,j−νmm,

where *D*_*V*_, *D*_*O*_, *D*_*p*_, and *D*_*m*_ are diffusion coefficients, *α*_*V*_, *α*_*p*_, and *α*_*m*_ are production rates, and *ν*_*V*_, *ν*_*p*_, and *ν*_*m*_ are decay rates for the related species; *λ*_*V*_ is the consumption rate of growth factors by endothelial cells and by unit length of vessels; *γ*_*v*_ is the permeability coefficient of the vessels; *O*_*v*_ is the intravascular concentration of oxygen, taken as a constant; *V*_max_ is the maximum uptake of growth factors when all cell receptors are saturated. The coefficient *k*_*i*,*j*_ is the uptake rate of oxygen which depends on the cell state in element (*i*, *j*): if (*i*, *j*) ∈ *P*, *k*_*i*,*j*_ = *k*_*P*_ (proliferative cells), if (*i*, *j*) ∈ *Q*, *k*_*i*,*j*_ = *k*_*Q*_ (quiescent cells), and if (*i*, *j*) ∈ *A* or *N* (apoptotic or necrotic dead cells), *k*_*i*,*j*_ = 0, else *k*_*i*,*j*_ = *k*_0_ (default uptake rate associated with normal cells) with *k*_*P*_ ≥ *k*_0_ ≥ *k*_*Q*_. *n*_*i*,*j*_ represents an angiogenic sprout (endothelial cells) located on one edge of the grid element (*i*, *j*). The cells forming the sprouts have an intense proteolytic activity,* via* the production of proteases that degrade the matrix to ease cell migration. All the parameter values are given in Table 1.

### 2.3. Model Initialization

**Figure 2:**
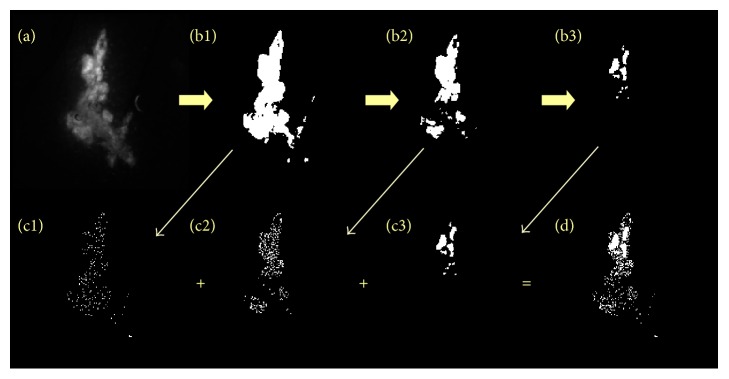
Stages of the procedure to transpose the fluorescence information of tumour cells to the computational framework. (a) Original GFP fluorescence image; (b1), (b2), and (b3) successive threshold images; (c1), (c2), and (c3) corresponding noised images; and (d) reconstructed final image obtained by summing images (c1), (c2), and (c3). The procedure is illustrated here on tumour case M4-L.

A virtual clone for each tumour is built from the extraction of structural elements: shape and density of the tumour, local vascular structure. Those are obtained by segmenting the images acquired immediately after the tumour cells injection (or no later than 3 days after). The GFP fluorescence image is used to extract the tumour cells repartition and the information on cell density is captured by the level of fluorescence intensity. Three intensity thresholds for the fluorescence are applied successively on the original image (Figure 2(a)) to differentiate three regions with various cell density from low density region to high density region (Figures 2(b1) to 2(b3)). The images are resized to the size of the computation grid (200 × 200 pixels). We then apply to each identified region some Gaussian noise (grey levels) and the images are filtered again with three thresholds to reflect the three different levels of cell density in each region (Figures 2(c1) to 2(c3)). The three resulting images are added to produce the initial virtual tumour (Figure 2(d)). We note that the thresholds applied are not necessarily the same for all the tumours since the fluorescence intensity varies from one tumour to another. They are chosen empirically with the aim to distinguish 3 levels of densities. In a future stage of development we intend to standardize the procedure.

**Figure 3:**
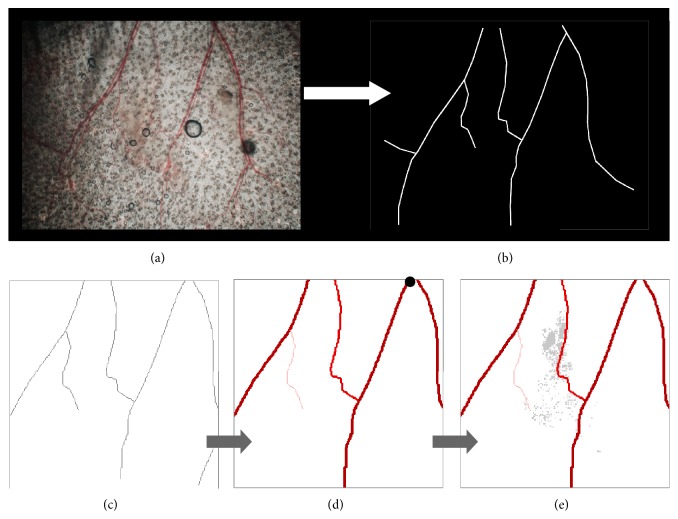
Stages of the procedure to transpose the bright field image information of the vascular network into the computational framework. (a) Bright field image of the vasculature; (b) the main vessels segmented; (c) the image cropped to the region of interest; (d) the vascular network perfused at the entry point highlighted by the black dot and vascular adaptation simulated until a stationary state is reached; (e) the resulting adapted vasculature merged with the extracted information on the tumour cells distribution. The procedure is illustrated here on tumour case M4-L.

The vessels of the vascular network with a diameter of at least 30 *μ*m are segmented and their coordinates are transposed in the model. Very often, venules and arterioles are parallel to one another. Since we do not distinguish them in our computational framework, only one of the two is represented in that case. Only the vessels next to the tumour are taken into account. The segmented image is cropped and resized to the size of the computation grid (200 × 200 pixels) (Figures 3(a) and 3(b)). A reference vessel is chosen and its diameter is set to 80 *μ*m; all the other vessels are initially assigned a diameter of 30 *μ*m. A pressure of 13 kPa is imposed at the entry point of the reference vessel (black dot in Figure 3(c)). The pressure at the boundaries of the domain is set to 2 kPa. Vascular adaptation under the effects of hemodynamical constraints induced by the blood flow is simulated according to the model presented in [31]. All vessels are free to adapt until a steady state is reached, except the reference vessel of which diameter is fixed to ensure stability of the whole network (Figures 3(c) and 3(d)). The resulting vasculature is then used as the initial vascular condition for tumour growth.

## 3. Results

### 3.1. Tumour Growth through Texture and Size

**Figure 4:**
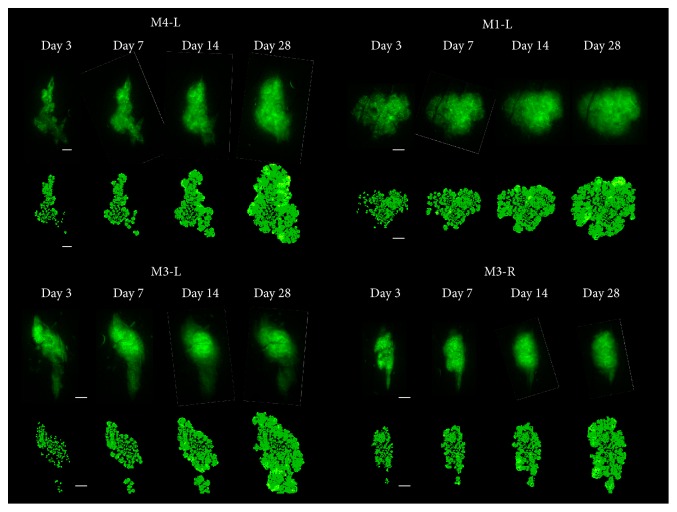
GFP fluorescence images (upper row in each frame) are compared with snapshots of the simulations (lower row in each frame) taken at four time points (days 3, 7, 14, and 28) for four different tumour cases (M4-L, M1-L, M3-L, and M3-R). Colour code for the simulations: proliferative cells in light green, quiescent cells in green, and apoptotic cells in yellow. Scale bars: 500 *μ*m. The corresponding simulations are available in Supplementary Data Video in Supplementary Material available online at http://dx.doi.org/10.1155/2016/7851789.

**Figure 5:**
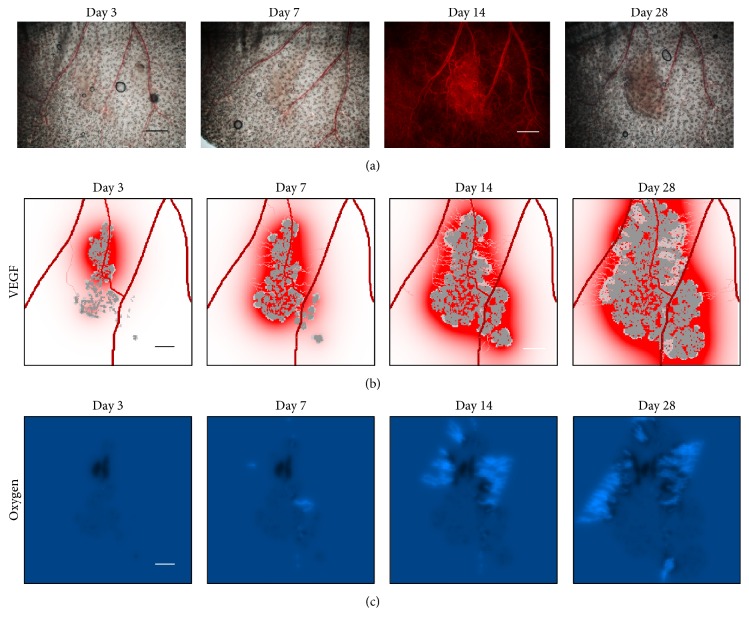
Comparison of the growth evolution of a simulated tumour (tumour case M4-L) with its real counterpart at four time points (days 3, 7, 14, and 28). (a) Bright field images of the tumour with 2x objective and RFP-fluorescence image of the vasculature (on day 14); (b) associated virtual tumour exhibiting the tumour cells (proliferative cells in light grey and quiescent cells in grey), vessels (darker vessels are larger), and VEGF distribution (in red); (c) oxygen level (dark spots for oxygen levels lower than normal and bright spots for oxygen levels higher than normal). Scale bars: 1 mm for experimental images (a); 500 *μm* for simulated images (b, c).

Tumour development is followed using intravital fluorescence imaging. The different imaging modalities give access to detailed and specific structural information on the tumour and its vasculature (Figure 1). The microscopic structural information is then integrated to build up the virtual tumour (Figures 2 and 3). Real tumour development is followed over 28 days and the comparison with the parameterized virtual counterpart is made at 4 discrete time points (days 3, 7, 14, and 28). Four different cases are presented in Figure 4. Simulation results are presented in order to provide graphical representations compatible with experimental observations. Fluorescence images which reveal tumour cells are compared with simulations only showing the tumour cells while distinguishing the different cell states: proliferative, hypoxic, and dying cells. Brighter zones in the experimental images correspond to higher tumour cell densities with proliferating cells. Similarly in the simulation, proliferative cells are represented with a brighter colour. Experimental bright field images show both the vascular network and the tumour mass (Figure 5(a)). The corresponding simulations exhibit the vascular network and the distribution of growth factors (VEGF) produced by the hypoxic tumour cells (Figure 5(b)).

Since all cells are initially introduced with a proliferative state (in the computational model), they almost immediately exhaust the oxygen resource by consuming oxygen and by degrading the underlying capillary network field [29, 30, 34] since the introduced tumour mass is important. The tumour cells become hypoxic and turn into a quiescent state. We note that in our model hypoxic and quiescent cells are in fact the same cells. The hypoxic cells release growth factors (such as vascular endothelial growth factor, VEGF). Depending on the location of the closest vessels, angiogenesis will start more or less rapidly to bring back oxygen to the hypoxic tissue (Figure 5(c)). Tumour cells in the vicinity to the newly formed vessels will turn back to the proliferative state and will primarily fill the empty gaps between cells, thus increasing the tumour density and allowing the tumour to grow.

Similarly in vivo, we observe that tumours become more compact with time. Compaction can be qualitatively assessed from the texture of the GFP fluorescence images (Figure 4) and the size of the tumours. On day 3, the fluorescent image is very granular,* that is, *heterogeneous which allows us to differentiate dense regions corresponding to higher fluorescence intensity from low density regions with weaker signal intensity. With time, the image texture becomes smoother, which reveals that cell proliferation and movements homogenize the cells spatial distribution.

**Figure 6:**
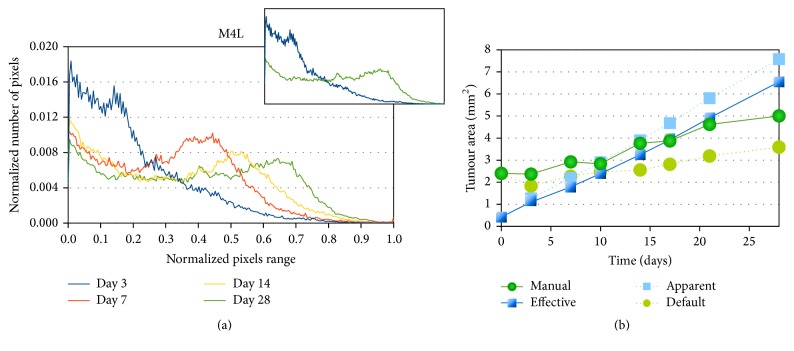
Tumour evolution (case M4-L). (a) The evolution of the tumour fluorescence distribution is assessed over 4 time points (days 3, 7, 14, and 28). The horizontal axis represents the fluorescence intensity of the images from dark pixels (with value 0) to bright pixels (with value 1). The range of pixel intensity values has been normalized for each image between the minimum value (darker pixel equals 0) and the maximum value (brighter pixel equals 1) since they are not necessarily the same from one image to another. The vertical axis stands for the number of pixels for each fluorescence intensity normalized by the total number of tumour pixels (the integral of each curve equals 1). The close-up compares day 3 with day 28 to exhibit the switch in the tumour fluorescence profile. (b) The evolution of the tumour area is first evaluated from the experimental images (green curves with bullets) using both a manual Yen-guided filter (plain line) and the Default ImageJ filter (dotted line). The curves are then compared with the areas measured on the corresponding virtual tumour (blue curves with squares), where the effective area is the area effectively occupied by the tumour cells (plain line) and the apparent area is the area which is delineated by the tumour's edge (dotted line).

The changes in the texture on the tumour fluorescence images from day 3 to day 28 are quantified and characterized from the distribution curves of fluorescence intensity. To that end, the fluorescence intensity and the intensity range have been normalized to make the comparison possible since the intensity levels are different from one image to another depending on how the image has been tuned to avoid pixel saturation. The resulting curve corresponding to the previously presented tumour is displayed in Figure 6(a). We recall that only pixels associated with the tumour are taken into account. On day 3, darker pixels dominate; the distribution of fluorescence intensity is heterogeneous and decreases sharply leaving very few bright pixels. This gives a rough (granular) texture. On the subsequent days, the fluorescence distribution becomes more homogeneous with less darker pixels and significantly more brighter ones, which gives a smoother texture. The close-up in the figure compares day 3 with day 28 to highlight the signal transition. The progression with time of this transition is particularly clear for this tumour case (Figure 6(a)) with a significant increase of bright pixels.

In the meantime, the tumour size which is estimated from its apparent area is multiplied in average by 1.67 ± 0.36 in 28 days (by 1.25 for tumour case M1-L and by 2.08 for tumour case M4-L, which are, resp, the slowest and the fastest growth on our group of tumours). The apparent area taken alone is however not sufficient to assess correctly the tumour growth in the two-dimensional plane, that is, the increase of the number of tumour cells in this plane. Correlation with the granularity is necessary to obtain a better estimation related to tumour cells density. A qualitative good match is attained between the real tumour and the virtual one based on three criteria: the shape, size, and texture.

### 3.2. Growth Kinetics and Limitations

Comparisons of the real and virtual (simulated) tumour growth are realised from the evaluation of the tumour area at discrete time points. Experimentally the tumour area is measured from the fluorescence images and depends on the filter used to segment the image (see Image Analysis). For the virtual tumour, the area can be directly calculated from the number of cells since each cell occupies one element of the cellular automaton. This gives us the effective tumour area. However we also estimate the apparent tumour area which is obtained by delineating the tumour edge. The different curves for the evolution of the tumour area with time are plotted on a same graph in Figure 6(b). These curves are the two experimental curves which correspond to two different filters: the restrictive one gives the lower estimation for the tumour area whereas the other one is more tolerant, so artefacts are manually corrected which gives a more accurate estimation; the two theoretical curves which correspond to the effective and apparent areas.

Figure 6(b) shows some discrepancies between the curves (which highlights the limit of the model). First, there is a difference at time *t* = 0. The virtual tumour areas are smaller than the experimental ones. This is due to the strategy we employ to define the initial virtual tumour. Only the bulk of the tumour is taken into account for the estimation of the tumour area. The scattered cells are not taken into account (see Figure 2(d)) whereas in the experimental case, the integration of other plans (third dimension) allows the detection of a larger tumour surface. There is a relatively good fit between the virtual and real tumours from day 7 to day 21: the measured virtual tumour area is catching up with the estimated real one since the virtual tumour cells fill the gaps in the simulated 2D plan. They further develop after the onset of angiogenesis. Some divergence can occur more or less rapidly above 21 days. Those are especially visible in the tumour case presented in Figure 6(b) where the virtual tumour expansion is faster than for the real tumour. This is once again related to the different dimensionality of the virtual (2D) and real (3D) tumours but also to a compaction effect that has been disregarded in the virtual tumour model due to the fact that there can only be one cell per element of the automaton.

### 3.3. The Angiogenic Bottleneck

**Figure 7:**
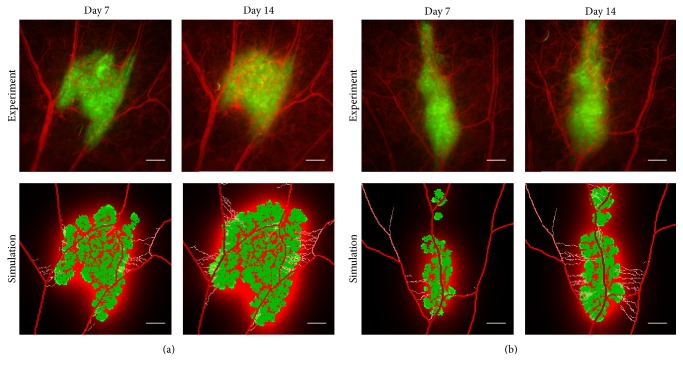
Tumour and vascular states are compared between day 7 and day 14 for two tumour cases: M2-R (a) and M4-R (b). In experimental images, the vasculature is highlighted in red using dextran-rhodamine and the U87-GFP tumour cells are highlighted in green. In the simulated images, a corresponding colour code has been used. The vessels are in red and the neovessels in lighter red. The tumour cells appear in green with actively proliferating cells in brighter green. In the simulated images, the growth factors produced by the tumour cells appear in red in the background. Scale bars: 500 *μ*m.

The best match in terms of growth between the virtual tumour and its real counterpart is obtained from day 7 to day 21.  Figure 7 compares images that merge the two fluorescent channels with simulated images for two other tumour cases (M2-R and M4-R). The simulated images show the tumour, the vasculature, and the growth factors secreted by the hypoxic tumour cells. From the experimental images we observe that the background is darker at day 7 and much brighter at day 14. This is due to some leakage of the fluorescent dye (dextran-rhodamine) into the extravascular space induced by the growth factors which breaches the vascular walls as endothelial cells detached to form the angiogenic sprouts. This effects is indirectly captured in the simulated images where the increased growth factor concentration is related to the increase vessel leakage.

**Figure 8:**
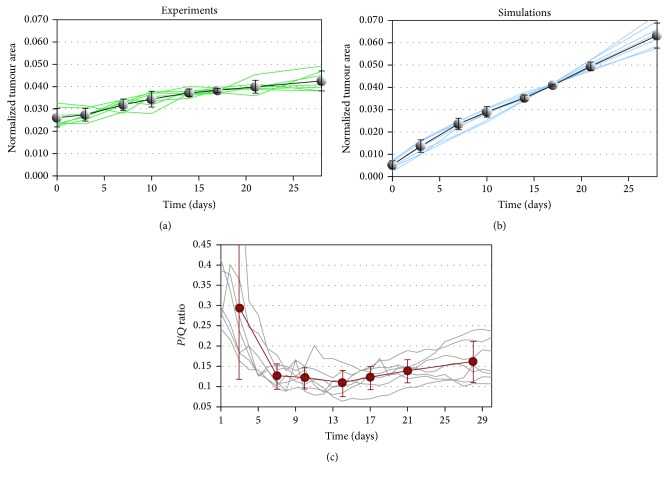
Tumour growth is monitored from the normalized tumour area in experimental images (a) and in the simulations (b). All tumour cases are represented in both graphics (curves in green for the experiments in panel (a) and in blue for the simulations in panel (b)) and the black curves correspond to the mean curves featuring the standard deviation for each point. (c) shows the evolution of the ratio between actively proliferating cells (*P*) and quiescent cells (*Q*) in the simulations for all the tumour cases (where each grey curve represents one tumour case) and the red curve represents the mean curve with standard deviation of the mean for each point.

Experimental and simulated growth curves of all the tumour cases are presented in Figures 8(a) and 8(b), respectively. The curves *s*_*k*_(*t*) (*k* = 1 to 7) have been normalized by their integral value,* that is,* $\tilde{s}(t)=s_{k}(t)/\int_{0}^{28}s_{k}(t)dt$, to make them all comparable. Experimental curves correspond to the manually determined tumour area of Figure 6(b). Simulated growth curves plot the evolution of the number of tumour cells which corresponds to the effective tumour area of Figure 6(b). Although the simulated growth curves are obviously more homogeneous than the experimental ones, there is a striking resemblance at day 17 where the curves variability is minimum for both experimental and simulated curves.

This corresponds to the well-known angiogenic switch [15] that we designate here by the term* bottleneck* that more accurately describes the observe phenomena by which slow growing tumours (slower than the average) have a higher angiogenic potential since they possess a higher proportion of hypoxic cells (producing growth factors), whereas faster growing tumours (faster than the average) produce a lower angiogenic response. As a result, the growth curves coincide at a specific time period due to a progressive and adapted angiogenic regulation which starts to develop after about a week and reaches its full capacity about 10 days later, leading to the emerging convergence of the normalized growth curves on day 17 (with the approximation of time sampling for image acquisition).

The proportion of quiescent cells (*i.e*., hypoxic cells) in the simulated tumours is highlighted in Figure 8(c). The tumour cells are initially (day 0) all proliferative. On day 3, only 30% in average are still proliferative; all other cells turned quiescent. In average the proportion of proliferative cells decrease to a minimum close to 10% on day 13. Angiogenesis then starts to produce some sensible effects on the cell population by reverting quiescent cells into a proliferative state. This leads to an increase proportion of proliferative cells visible from day 17 which confirms the interpretation of the angiogenic bottleneck effect by which tumour growth is progressively resumed.

## 4. Discussion

In this study we developed a model for tumour growth and angiogenesis that has been applied to build up a virtual clone of a real tumour. The model successfully describes the development of seven different tumour cases over a period of about a month, without requiring any changes or adjustments in the model parameters from one tumour to another. This shows that the model with the default set of parameters (Table 1), mostly taken from the literature and adjusted from a previous study [17], is robust. Interestingly, we identified what we called an* angiogenic bottleneck* characterizing the tumour development. This effect, observable from the experimental tumour growth curves, is very well captured by the computational model since it is found to be significantly emphasized in the simulated curves. This angiogenic bottleneck marks the convergence of the normalized growth curves around day 17 (Figure 8). It can be interpreted as a signature of* the progressive transition*—hence* bottleneck* rather than* switch*—between avascular and vascular (*i.e.*, angiogenic) tumour growth over this specific time period for all the tumour cases. We note that this phenomenon appeared in the simulations as an emerging property of the physiological model. It shows that the model, although simple, is able to catch a major characteristic of the experimental model. Specifically, the basic mechanisms for cell growth activation and inhibition through the regulation of the oxygen level and the mediation of growth factors are sufficient to reproduce the characteristics of tumour development in terms of shape, size, and density. The vascular structure of the microenvironment influences the cell shape and the tumour heterogeneity (active proliferating zones* versus* dormant quiescent zones) through its angiogenic potential stimulated by the tumour itself.

Although successful in catching key aspects of reality, the physiological model should be further improved to become reliable on a longer time scale. First the computational model can be generalized in 3D to exploit the potential of two-photon imaging that gives access to a tissue depth of a few hundreds microns for the reconstruction of a large tumour volume. Second, cell compaction that has been overlooked in this version of the model can be easily integrated in the computational framework by allowing more than one cell in a grid element. The consequences of the increase mechanical pressure on the modulation of the cell proliferation rate [35] and on vascular shutdown [34] can thus be described. This will allow us to account for the increased density of the tumour mass (by exploiting the image texture, rather than the tumour area) in order to match more accurately the tumour growth curve beyond the angiogenic bottleneck.

With this study, we have been able to show that a biological object, as complex as a tumour, can be transposed into a virtual clone to predict its overall behaviour. But the main interest in detaining such a reliable tool is its potential to investigate and predict the effects of therapies. Its main object is to use it as a virtual substitute to test a panel of therapeutic protocols (*i.e.*, by defining the drug dose, administration duration, and frequency). This is even more helpful in combined therapy protocols that may influence each other like the use of antivascular and cytotoxic drugs. This should help to identify the optimum therapeutic strategy for the real tumour. It is expected that such a personalized treatment, which takes into account all the tissue specificities of the patient (tumour shape and density and vascular configuration), would considerably increase its efficacy. Although precision medicine has been recently promoted and advertised through the rise of big data [3–6], we remain convinced that Personalized Medicine, involving biologically based computer models, is equally reachable and achievable.

## 5. Conclusions

Personalized Medicine is pursued as a major goal to fight cancer and requires the assistance of biologically based theoretical models. However deriving such highly informed and dedicated models is not easy. In this study we developed a virtual tumour based on basic mechanisms for cell growth activation and inhibition through the regulation of the oxygen level and the mediation of growth factors. Those mechanisms appeared to be sufficient to reproduce the characteristics of tumour development in terms of size and shape over a month. Moreover, the key angiogenic transition in the growth process was very well captured by the model, by spontaneously emerging in the simulations as a consequence of these simple physiological rules.

## Supplementary Material

Simulation of tumour growth over 28 days corresponding to the experimental cas M4-L displayed in figure 4.

## Figures and Tables

**Table 1:** Model parameters.

Parameter	Unit	Value	Description	Reference
*D*_*m*_	mm^2^·h^−1^	10.4 × 10^−3^	Proteases diffusion coefficient	[17]
*α*_*m*_	h^−1^	130	Proteases production rate by sprout	[17]
*ν*_*m*_	h^−1^	1.30	Proteases decay rate	[17]

*D*_*p*_	mm^2^·h^−1^	1.73 × 10^−3^	Tumour proteases diffusion coefficient	Adapted from [17]
*α*_*p*_	h^−1^	3600	Proteases production rate by tumour cells	Adapted from [17]
*ν*_*p*_	h^−1^	0.21	Tumour proteases decay rate	Adapted from [17]

*D*_*V*_	mm^2^·h^−1^	0.104	Growth factor diffusion coefficient	[18]
*α*_*V*_	s^−1^	0.0145	Growth factor production rate by quiescent cells	Estimated
*ν*_*V*_	h^−1^	0.65	Growth factor decay rate	[19]
*λ*_*V*_	h^−1^	1	Consumption rate of growth factor by endothelial cells	[19]
*V*_max_	h^−1^	0.06	Max consumption of growth factor by endothelial cells	[19]

*D*_*O*_	mm^2^·h^−1^	2.41 × 10^−3^	Oxygen diffusion coefficient	[20]
*γ*_*p*_	mm^−1^·s^−1^	4.8 × *R*/*R*_min_	Vessels permeability to oxygen	[21]
*k*_0_	s^−1^	2.4	Oxygen consumption rate by normal cells	Based on [22]
*k*_*P*_	s^−1^	2*k*_0_	Oxygen consumption rate by proliferative cells	Based on [21]
*k*_*Q*_	s^−1^	*k*_0_	Oxygen consumption rate by quiescent cells	Based on [21]
